# Social Media Algorithms and Teen Addiction: Neurophysiological Impact and Ethical Considerations

**DOI:** 10.7759/cureus.77145

**Published:** 2025-01-08

**Authors:** Debasmita De, Mazen El Jamal, Eda Aydemir, Anika Khera

**Affiliations:** 1 Neonatology, Vanderbilt University Medical Center (Former), Nashville, USA; 2 Medical Sciences, Lebanese University, Beirut, LBN; 3 Medicine, Ankara Yıldırım Beyazıt University, Ankara, TUR; 4 Medicine, BASIS Scottsdale High School, Scottsdale, USA

**Keywords:** cognitive functions, ethics, machine learning, reward system, social media addiction

## Abstract

Does it matter how many hours we spend scrolling through Instagram? This article examines the neurobiological impact of prolonged social media use, focusing on how it affects the brain's reward, attention, and emotional regulation systems. Frequent engagement with social media platforms alters dopamine pathways, a critical component in reward processing, fostering dependency analogous to substance addiction. Furthermore, changes in brain activity within the prefrontal cortex and amygdala suggest increased emotional sensitivity and compromised decision-making abilities. The role of artificial intelligence (AI) in this process is significant. AI-driven social media algorithms are designed solely to capture our attention for profit without prioritizing ethical concerns, personalizing content, and enhancing user engagement by continuously tailoring feeds to individual preferences. These adaptive algorithms are designed to maximize screen time, thereby deepening the activation of the brain's reward centers. This cycle of optimized content and heightened engagement accelerates the development of addictive behaviors. The interplay between altered brain physiology and AI-driven content optimization creates a feedback loop that promotes social media addiction among teenagers. This raises significant ethical concerns regarding privacy and the promotion of personalized content. This review article offers a comprehensive and in-depth analysis of the neurophysiological impact of social media on adolescents and the moral concerns governing them. It also provides solutions for ethical social media use and preventing addiction among teenagers.

## Introduction and background

Social media has become a global phenomenon driven by rapid expansions in Facebook, Instagram, YouTube, Snapchat, and TikTok. In 2024, the number of active social media users worldwide has surpassed 5 billion and is projected to reach over 6 billion by 2028 [1]. Most disturbingly, however, it is the sudden increase in social media addiction that affects the most vulnerable teenagers and leaves them most prone to its negative influences. Studies have shown that almost a third of all social media users are adolescents and young adults, while 93-97% of all teenagers aged 13 to 17 years use at least one form of social media [2,3]. Of these, adolescent girls aged 16 to 24 years spend more than three hours daily on social media, while for boys of the same age group, it is approximately two and a half hours, hence becoming the most active users in that age group for any demographic evidence [2]. Social media use has become an integral part of adolescents' lives. It holds the lion's share in affecting adolescents' perception of peer acceptance and social identity [4]. Moreover, the lack of parental supervision on these sites gives the teens more leeway to satisfy these psychosocial needs [5]. The growing concerns have equally seen a coalition of 41 states and the District of Columbia filed a lawsuit against Meta, accusing it of intentionally designing addictive features aimed at kids despite assurances that the sites are safe for younger users. This situation raises key questions about why teens get hooked on social media, potentially adversely affecting brain development and overall functioning.

Several terminologies have been used to define social media addiction, including problematic social media use (PMSU) [6], problematic social networking site use (PSNSU) [7], social media disorder [8], Facebook addiction [9], and Facebook dependence [10], within the last couple of years by researchers. Yet, a common consensus on its definition remains a far cry. Generally, social media addiction has been defined as excessive and compulsive use, which can be characterized by an uncontrollable urge to browse social networking sites [11] constantly. A few researchers have broadened the concept to include both internet addiction (IA) and smartphone addiction (SPA) under the heading of social media addiction. IA is defined as the inability to control the use of the internet and computers interfering with the daily life of a person linked to anxiety, depression, attention deficit hyperactivity disorder (ADHD), stress, low self-esteem, and diminished psychological well-being [12]. Similarly, SPA is known as excessive utilization of smartphones that interferes with daily activities and primarily creates anxiety and anger, social isolation, etc. [12]. Despite these diverse terms and conceptualizations, there is no consensus on what constitutes a definition of social media addiction. Moreover, except for internet gaming disorder, social media addiction has not yet been listed as a standard diagnosis in either the Diagnostic and Statistical Manual of Mental Disorders, Fifth Edition (DSM-5) or the International Classification of Diseases, 11th Revision (ICD-11) [7,13].

The problem of social media addiction is growing day by day with the widespread use of smartphones and platforms like Facebook, Instagram, Snapchat, TikTok, and Threads. These platforms use frequent updates, notifications, and endless scrolling feeds that distract users, shift focus from essential tasks, and create a state of partial attention [14]. As highlighted in an article by GeeksforGeeks, advanced machine learning algorithms such as natural language processing, linear regression, and clustering analyze user behavior to interpret sentiments and interests. This enables platforms to recommend tailored content and optimize real-time feeds, increasing user engagement and fostering addictive behaviors [15]. The impact is particularly concerning for adolescents, who are highly susceptible to compulsive internet use due to their developmental struggles with perceptual awareness [16]. During this critical phase, adolescents undergo significant behavioral changes and exhibit heightened sensitivity to rewards, making them especially vulnerable to IA and its consequences [17].

Besides addiction, social media algorithms that incorporate artificial intelligence (AI) technology also raise significant ethical concerns, particularly for teenagers. These platforms are fully committed to maximizing profits by pleasing the advertising companies, which target specific demographics by creating continuous feeds that keep users on their platforms for as long as possible [18]. In 2022 alone, US children aged 0 to 17 years generated advertising revenues of $11 billion for major social media platforms [18]. Tim Estes labeled AI "fentanyl" when added to the "digital heroin" of social media in Newsweek [19]. Tore on browsing habits or even "private conversations," the AI-powered algorithms would then push targeted content: harmful diet strategies by influencers, deepfake videos, extremist material, or content dealing with mental health. Inevitably, that only feeds anxieties and turbocharges anxiety and depression [19]. A meta-analysis further ascertained that there is a linear dose-response relationship between time spent on social media and the risk of depression. Among adolescents, it found a 13% increase in the incidence of depression for every additional hour spent on social media [20]. Against this pervasive rise in social media use among teens (96% of adolescents in Canada use social media, while 7.38% of European adolescents and 4.5% of Hungarian adolescents were identified with problematic usage [21]), it is essential to understand the adverse impacts of social media on teens and their brains that are under development.

Getting more profound insights into its impact means higher chances of discussing and taking interventions based on well-informed perspectives. This article discusses how social media serves as a vector for addictive behaviors by utilizing the brain's reward system. It addresses changes it may cause in the structure and cognitive functions of the brain. It also focuses on some ethical issues related to social networking and the intervention methods that can be used to prevent these adverse effects.

## Review

How the reward system of the brain fosters social media addiction

**Figure 1:**
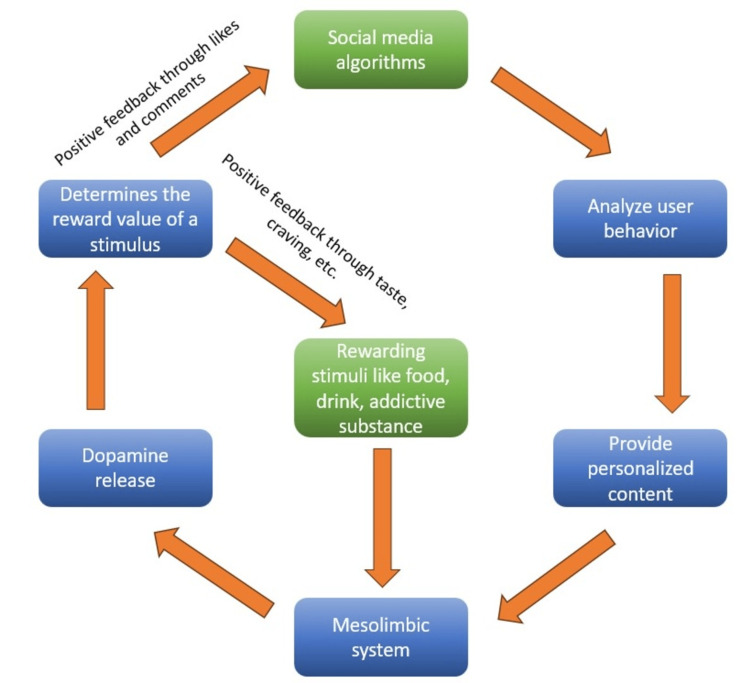
How the reward system of the brain fosters social media addiction. Figure credit: Debasmita De.

The mesolimbic system, also referred to as the brain's reward system, controls the processing of rewards by associating specific stimuli with positive outcomes, thus influencing behavior. Dopamine is considered to be at the core of this process, determining the reward value of experiences such as food, sex, and social interactions [22]. Upon exposure to rewarding stimuli, the mesolimbic system releases dopamine into specific target nuclei [23,24]. Drugs of abuse are known to stimulate the dopaminergic mesolimbic pathway that includes the ventral tegmental area and nucleus accumbens, leading to addiction [25]. There is evidence that all addictions, both drug-related and behavioral, result from a common mechanism in the brain [26]. Social media systems are taking advantage of the system by increasing dopamine release via digital footprints and machine learning algorithms that flash personalized content. This reinforcement motivates extended use, while users find it harder to unplug due to the expectation of rewards [27]. Adolescents may be particularly vulnerable to online gaming addiction due to genetic variations, such as those in genes encoding dopamine D2 receptors and dopamine-degrading enzymes, which increase susceptibility [28]. They are often victims of an unrelenting "dopamine cycle" created in a loop of "desire" induced by endless social media feeds, "seeking and anticipating rewards" in the way of photo tagging, likes, and comments, the latter being the triggers that continue to reinstate the "desire" behavior. The overactivation of the dopamine system in such individuals can further increase the risk of addictive behaviors or pathological changes that lead to a decline in pleasure from natural rewards; this is what is referred to as reduced reward sensitivity, a hallmark of addiction (Figure 1) [29].

Impact of social media addiction on the structure and cognitive functions of the brain

Structurally, the basal ganglia, amygdala, and prefrontal cortex are critical in developing and maintaining addictive behaviors. Dysregulation in these three regions can enhance the focus on incentives and weaken executive controls such as decision-making and regulation of actions, emotions, and impulses [30]. Internet addiction has increased grey matter volume in the bilateral putamen and right nucleus accumbens and decreased grey matter volume in the orbitofrontal cortex, a part of the prefrontal cortex [31]. It can also interfere with action plans, favoring immediate rewards over long-term benefits [32]. Moreover, smartphone addiction can reduce neural activity in the anterior cingulate cortex (ACC) [33].

**Figure 2:**
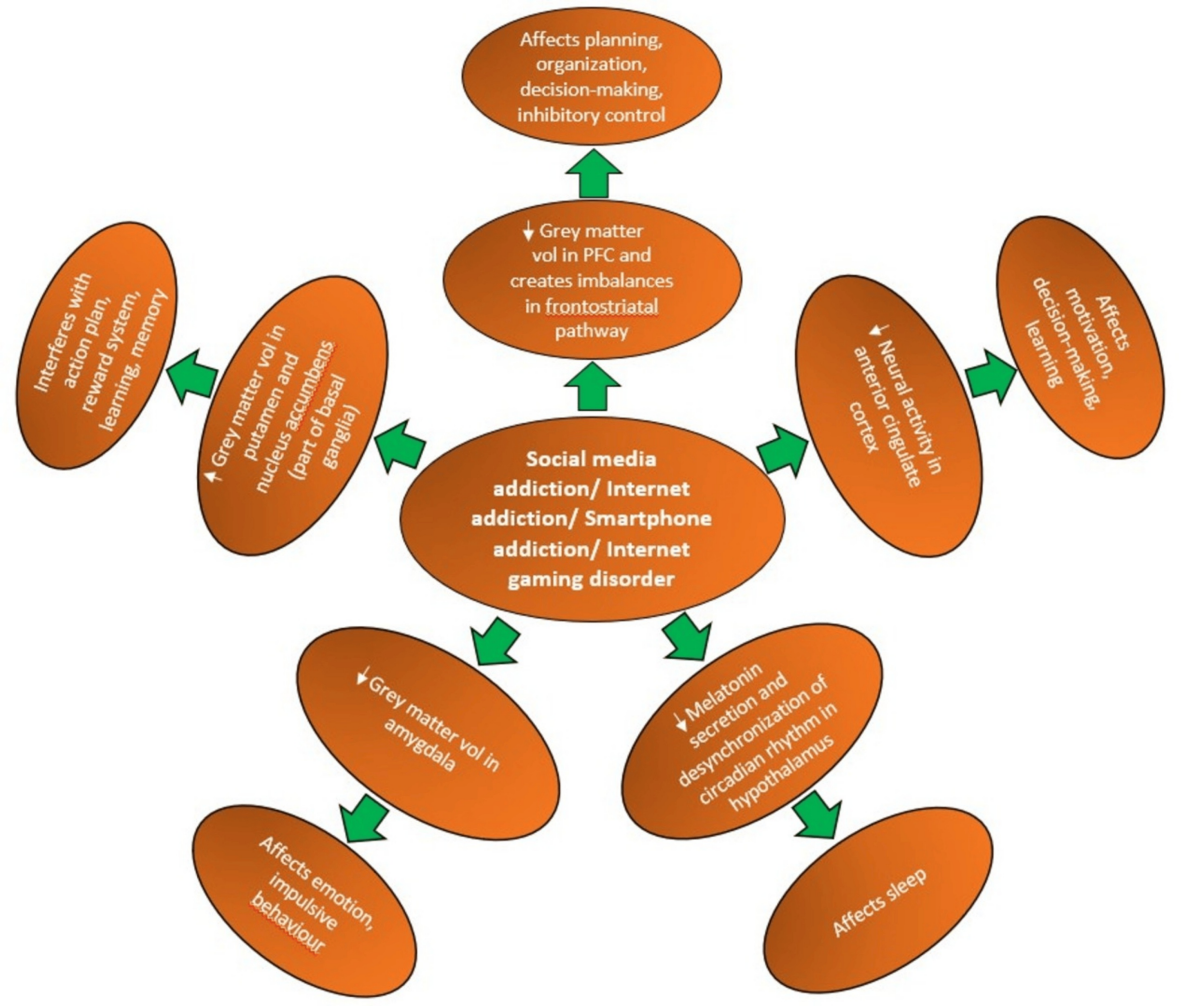
Different parts of the brain affected due to social media addiction. PFC: prefrontal cortex; vol: volume. Figure credit: Debasmita De.

The brain's cognitive functions embrace information processing, attention, working memory, structured search, internal modeling, and flexibility in thinking and actions. According to the Interaction of Person-Affect-Cognition-Execution (I-PACE) model, internet use affects attention, mood, emotional regulation, and inhibitory control involved in decision-making [34]. Internet addiction can also cause changes in the prefrontal cortex and lead to an imbalance in the frontostriatal pathway, which increases sensitivity to stimuli and reduces inhibitory control, thus influencing decision-making and emotional changes [35]. In adolescents and young adults, impairments in cognitive functions, such as self-monitoring, memory retention, organizational skills, and time management, are commonly seen in cases of internet and smartphone addiction [12]. Individuals with internet gaming disorders have been found to engage in higher risk-taking behaviors driven by dysregulation of the reward processing system, impaired impulse control, and weakened inhibitory mechanisms [36]. Studies have also found that adolescents who have the fear of missing out are more prone to social media addiction, which can significantly affect their sleep quality. This is further exacerbated by using portable smartphones before bed, reducing melatonin secretion and desynchronizing the hypothalamus's circadian rhythm [37]. Addiction to social networking sites can also reduce grey matter volume in the amygdala, which is related to emotion and strong impulsive behavior (Figure 2 and Table 1 show brain structures and related functions affected by social media addiction) [38]. Thus, it becomes clear that social media addiction has the potential to bring considerable changes in the structural and functional aspects of the brain among adolescents and young adults. The situation tends to get more alarming with rapid technological advancements and machine learning promoting social media addiction.

**Table 1:** Structure and the corresponding functions of the brain affected due to social media addiction.

Structure of the brain affected	Cognitive function affected
Prefrontal cortex	Higher-order decision-making, planning, organization, time management, and inhibitory control [39-41].
Basal ganglia	Reward processing, emotion, language, learning, and memory [42,43].
Amygdala	Controls impulsive behaviors and emotions [38,39].
Anterior cingulate cortex	Motivation, decision-making, and learning [44].

Ethical concerns that arise during social media use among teenagers

Above all, the most extensive usage of social media has raised several critical ethical issues regarding its addictive potential, privacy concerns, and the insufficiency of informed consent. These problems have become increasingly familiar with the increasing integration of social media into everyday life, especially for teenagers. Social media platforms are designed to offer constant stimulation and personalized content, which keep users hooked for extended periods [45,46]. As has been discussed earlier in this review, such designs nurture addictive behaviors that may cause mental health problems and reduced productivity. These risks raise serious safety concerns, especially for teenagers, who may be more vulnerable to the adverse effects of prolonged social media use.

Another big ethical issue is social media's large-scale collection of personal information. Personal details, browsing history, location, and purchasing habits are collected for targeted advertisements, content recommendations, and engagement maximization [46,47]. While these practices can enhance the user experience, they pose significant privacy risks. Many users, particularly teenagers, do not know the level to which data collected are put in service, even to influence them to change specific dimensions of their being and interactions. This element of less transparency increases the ethical concern more significantly. However, few consider the issue of informed consent. Primarily, social media firms do not inform the users about the risks associated with using social media and handling, storing, and utilizing the users' private data, mainly those below the user's age. They are to be provided in an easily understandable manner. Transparency by social media platforms shall empower users and help them know what is getting them into the usage.

Solutions and future directions: designing more ethical platforms

Since concerns over social media addiction are increasing, solutions must be sought and implemented. An excellent way to approach this issue is to prioritize user well-being over engagement period and profits. For example, social media platforms can build features to help users take breaks and avoid long screen time. Furthermore, social networking sites can start implementing new stratagems to let their users decide how they want to modulate their online experience: for instance, adding options in settings where users can select to see or skip certain kinds of content. Thus, the choice will come directly from the user. For instance, some users may choose to use the platforms for beneficial (e.g., educational) purposes without the need to see random fleeting trends. Concerning transparency, users should be informed in detail how their data are used and for what purposes. They should learn how algorithms work, and nothing should be concealed. The utmost priority must be users' well-being, not financial profits.

Limitations

Despite its strengths, there are several limitations. While the article has focused on neurobiological changes, it has not addressed how such effects might vary across different age groups, cultural contexts, or individual differences, like pre-existing mental health conditions. The focus on adolescents, while necessary, ignores other at-risk populations, such as young adults or those with predisposing psychological vulnerabilities. It also relies too much on secondary data and previously published studies, which might not consider the ever-changing nature of social media sites and algorithms. Longitudinal data are lacking; therefore, one cannot make any causal inferences about how social media addiction may influence changes in brain development over a long period. While very valuable to the insights it provides into the interplay between social media addiction, brain function, and ethical challenges, this review needs further research to address these limitations and provide a more holistic understanding of this pressing issue.

## Conclusions

Adolescents are prone to overuse of social media, leading to addiction, which is linked to depression and other detrimental health effects. Results show that lower levels of responsiveness in the ventral medial prefrontal cortex, medial prefrontal cortex, posterior cingulate cortex, and right inferior frontal gyrus throughout adolescence are linked to increased symptoms of addiction, such as social media use more than two years later. The association of social media users with symptoms of anxiety and depression has been well documented. Risk factors for this association include the quantity of time spent online, one's activity level, and social media addiction. Other research has looked at characteristics that may predispose people to social media use and addiction, in addition to evidence linking excessive social media use and addiction to psychiatric illnesses. ADHD and time spent on social media are major predictors of problematic internet use, reinforcing the idea that ADHD and social media addiction are linked. Despite this, minimal data on how social media neurologically impacts adolescents is available. This might be due to "addiction to social media" not being defined in the ICD and DSM as an "addiction" like other substance use disorders.

This review sheds light on the neurobiological impacts of prolonged social media usage, focusing on adolescents. A huge plus of the present study is its interdisciplinary nature, combining ideas of neurophysiology with those of machine learning algorithms toward unraveling the processes behind social network addiction. This, however, becomes much more important for the changes within the dopamine pathways and structural shifts in the prefrontal cortex and amygdala because of its vast effects on emotional control, decision-making, and processing rewards. Perhaps most saliently, this article elaborates on critical views about machine learning algorithms to show how such technologies affect changes in behavior - this is very contemporary. Besides, the article raises fundamental ethical issues regarding the role of social media platforms in fostering addiction, a strong foundation for future advocacy, and policy recommendations. The proposed intervention strategies, such as parental guidance, media literacy, and transparency in algorithmic operations, are actionable and forward-looking. Parents should be understanding, allow internet use in shared spaces but not bedrooms, disallow screen time during meals/sleep, encourage physical activities, and prevent social isolation. Additional recommendations include establishing peer-led support groups where teens can openly discuss social media use and share strategies for responsible engagement. Introducing media literacy classes or workshops as early as elementary school is also encouraged. A collaborative approach involving education, parental guidance, and transparency by tech companies is essential to fostering healthier social media habits and protecting the mental well-being of teenagers.
